# Black Races of Oceanica

**Published:** 1881-04

**Authors:** 


### THE BLACK RACES OF OCEANICA.

Negro forms are figured among the earliest representations of men on ancient monuments. As early as the eighteenth dynasty (seventeen hundred years before the Christian era), the artists of Egypt represented at least five races of negroes. Nigritic types were also figured by the Greeks, Romans, Assyrians, Babylonians and Persians, although none of those people had as extended knowledge of Africa as the Egyptians had. The examination of all the monuments which have come down from antiquity makes it evident that the negro races of Africa and Asia were well known. Scientific investigations of negro characteristics began to be made in the sixteenth century. The first to record one was Albert Durer, who, in 1525, drew a profile of a negro inclosed in a system of lines, of which an oblique and an horizontal line formed at their junction a real facial angle. MM. de Quatrefages and Hamy, in their "Crania Ethnica," begin the study of the negro races with the negroes of Oceanica, and select as their point of departure the Negritos, the most brachycephalic race. The Negrito race proper, which was first observed in the Philippine Islands, has been found in the interior of the Peninsula of Malacca, the Sunda Islands, and the Andaman Islands. M. Hamy has been able to trace it even to the interior of India.—DR. VERNEAU, *in Popular Science Monthly for April.*

